# Bioactive compounds, antioxidant activity, and mineral content of bróquil: A traditional crop of *Brassica oleracea* var. *italica*

**DOI:** 10.3389/fnut.2022.1006012

**Published:** 2023-01-10

**Authors:** Celia Montaner, Cristina Mallor, Sonia Laguna, Raquel Zufiaurre

**Affiliations:** ^1^Escuela Politécnica Superior de Huesca, Universidad de Zaragoza, Huesca, Spain; ^2^Instituto Agroalimentario de Aragón-IA2, Centro de Investigación y Tecnología Agroalimentaria de Aragón (CITA), Universidad de Zaragoza, Zaragoza, Spain; ^3^Departamento de Ciencia Vegetal, Centro de Investigación y Tecnología Agroalimentaria de Aragón (CITA), Zaragoza, Spain; ^4^Instituto Universitario de Investigación en Ciencias Ambientales de Aragón-IUCA, Universidad de Zaragoza, Zaragoza, Spain

**Keywords:** *Brassicaceae*, phytochemical analysis, nutraceutical, agrodiversity, landraces

## Abstract

*Brassicaceae* edible plants are rich in bioactive compounds and promote health benefits. However, there is less interest in expanding knowledge about the *Brassica* cultivars to date. In particular, underutilized species and local cultivars could constitute a source of agrodiversity in adapting to the territory with likely higher contents of nutraceutical compounds. In this context, Bróquil (*Brassica oleracea* var. *italica*) is a traditional *Brassicaceae* crop grown in the Spanish region of Aragón. Currently, it is cultivated mainly in family orchards for autoconsumption and, in minority, in small farms for local markets. This study evaluates a collection of 13 bróquil landraces from the Spanish Vegetable Genebank of the Agrifood Research and Technology Center of Zaragoza (BGHZ-CITA), describing their mineral contents, bioactive compounds, and antioxidant activities, including a broccoli commercial variety “Parthenon” as the control. The study reports data on the health-promoting nutrients and antioxidants of bróquil for the first time. Under our experimental conditions, we found that bróquil has a great variability for these compounds that showed on average similar or higher levels than the broccoli control. The different bróquil landraces also revealed variability in both intraccessions and interaccessions due to the lack of a formal breeding selection. Despite this variability, we highlight accession HB5 that corresponds to Headed Bróquil BGHZ6685. In particular, we can stand out its antioxidant activity of 87.07 ± 0.81%I, total phenolic content of 13.21 ± 0.53 mg GAE g^−1^ dw, total flavonoid content of 14.50 ± 1.29 mg QE g^−1^ dw, total glucosinolate content of 43.70 ± 1.09 mg SnE g^−1^ dw, and vitamin C content of 7.21 ± 0.13 mg AA g^−1^ dw. Regarding bróquil mineral composition, K was the highest macroelement (22.66–33.62 mg g^−1^ dw), followed by Ca, P, and S whose values were relatively lower compared to K. Mg and Na showed the lowest values. Among the microelements evaluated (Mn, Zn, and Fe), iron was the most abundant detected, higher in all bróquil accessions than in broccoli, except for one accession. Therefore, the results reported for bróquil landraces show promising nutritional quality. This could lead to an increase in agrobiodiversity and contribute to a more diversified and healthy diet.

## 1. Introduction

Vegetable crops represent a fundamental ingredient in the human diet due to their high nutritional value and bioactive content and could serve toward improving food security and nutritional quality (1). However, modern cultivars, due to their decreasing agrobiodiversity, exhibit reduced resilience to pests and climatic changes and may, in several cases, show lower levels of bioactive compounds (2). In contrast, primitive landraces developed by growers represent exciting and unexploited locally adapted germplasm. These landraces have been excellent sources of bioactive compounds in several vegetables as revealed by recent studies on carrots (3), tomatoes (4), lettuce (5), and pepper (6).

In the past decades, special attention has been paid to *Brassicaceae* edible plants due to their richness in bioactive compounds and health benefits (7, 8). Similarly, bróquil (*Brassica oleracea* L. var. *italica, Brassicaceae* family) is a traditional winter cole crop that has been cultivated in the Huesca Province of the Aragón region, Northeast Spain, since ancient times (9). As no commercial breeding varieties are available, the farmers have retained this landrace for generations, thus preserving the plant material. Currently, although bróquil production has been relegated to family orchards for autoconsumption, it is also cultivated in small farms that supply nearby regional markets. Bróquil is locally appreciated for its taste and peculiar flavor. It is an ingredient of typical dishes, and local chefs use this vegetable as the feedstock of avant-garde cuisine. The edible parts of the plants are a mix of young leaves and immature inflorescences, heads, or curds. A bróquil plant is formed by many sprouts born from older leaves' axillary buds. Each sprout is formed by a group of young leaves surrounding a small curd when the floral stage arrives. Two different types of bróquil plants are cultivated: the “green bróquil” type (locally known as “bróquil verde”) and the “headed bróquil” type (or “bróquil pellado”). The curds are bigger and predominate over leaves in the headed bróquil type. In contrast, leaves predominate over the curds in the green bróquil type. The color heads are yellow, from pale to yellowish green resembling cauliflower. The inflorescences of both bróquil types seem to be ontogenetically older than those of broccoli as in the case of cauliflower (10). Marketable maturity arrives when curds start to appear and form together with the leaves, a compact plant structure.

Local nursery owners multiply and maintain their own varieties by using cultural practices like other *Brassica* crops. In the traditional area, the growth cycle begins at the end of June. The harvesting time starts in December and lasts until March, so it is traditionally consumed at Christmas. The green bróquil cycle is shorter than that of headed bróquil, although this fact is dependent on plant genetics and environmental conditions. Entire plants need to achieve on average a weight of four kg for commercial maturity. External leaves are removed before marketing and around 10% of plant weight will be the raw ingredient for cooking (11). Hence, research is being carried out to take advantage of these byproducts (12). Currently, there are only four accessions of bróquil cultivation in farms, although the Spanish Vegetable Genebank of Zaragoza (BGHZ-CITA) has been maintaining an *ex-situ* collection of 13 accessions of bróquil that represents the variability of this landrace in the traditional growth area.

Numerous epidemiological studies indicated that *Brassica* vegetables and broccoli, in particular, protect humans against diseases as they are rich in glucosinolates and possess high contents of phenols, flavonoids, vitamins, and mineral nutrients (13–15). However, there is not much interest in expanding the knowledge about the characterization of the phytochemical compounds of *Brassica* cultivars, especially the local cultivars, as a source of agrodiversity adapted to the territory and with foreseeable higher contents of nutraceutical compounds. Less-known cultivars, such as bróquil, are of quite an interest to be studied to characterize them and find higher qualitative properties than other conventional *Brassica* cultivars. This could also be useful for consumers who could find new healthy vegetable products in the market (16). Therefore, the purpose of the current study was to evaluate the variability of the entire collection from the Spanish Vegetable Genebank of Zaragoza (BGHZ-CITA), consisting of 13 bróquil accessions, based on their mineral contents, bioactive compounds, and antioxidant activities.

## 2. Materials and methods

### 2.1. Plant materials and experimental design

**Figure 1:**
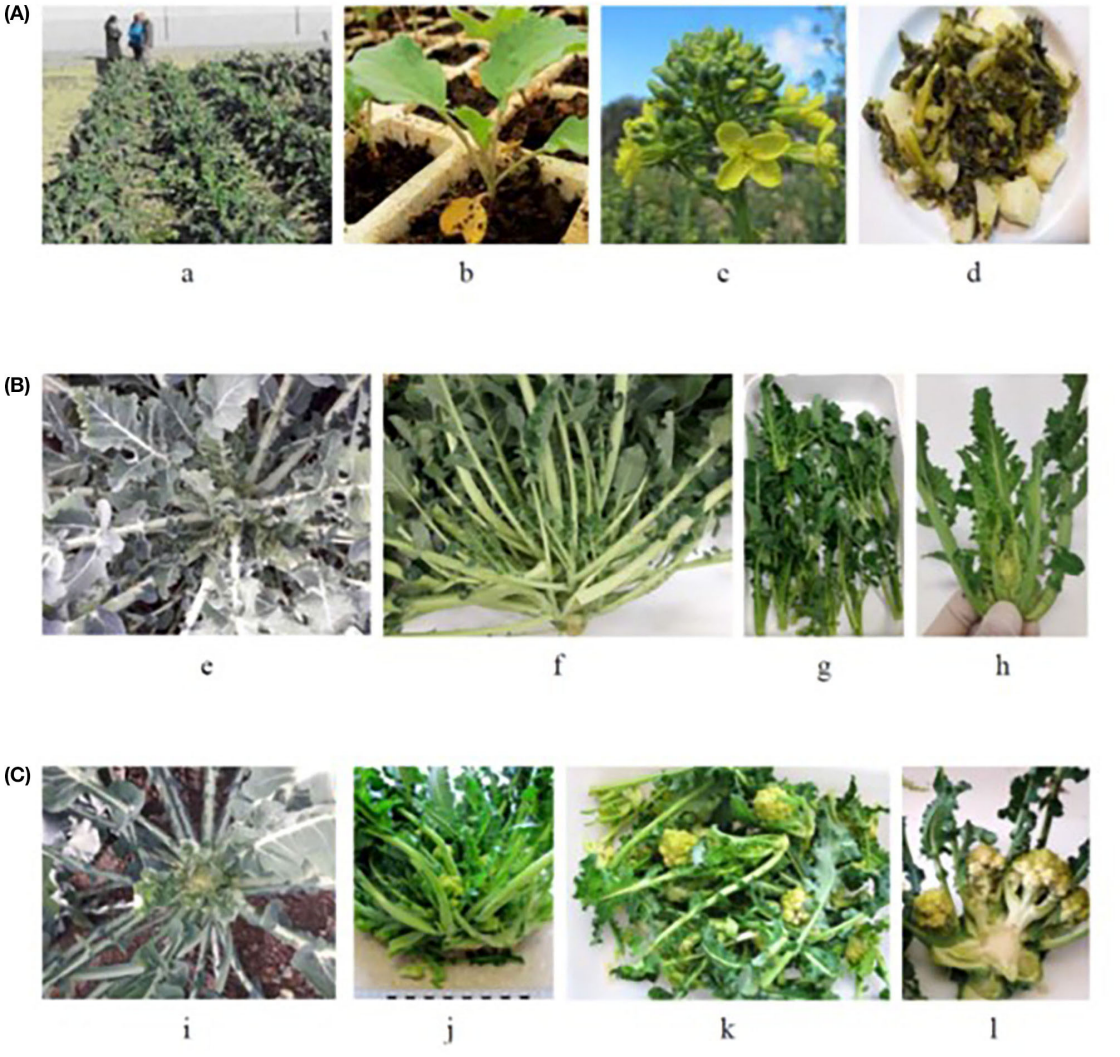
**(A)** Bróquil: (a) field, (b) seedling, (c) inflorescence, and flower (d) traditional cooking. **(B)** Green bróquil type: (e) plant in the field, (f) sprouts in the commercial stage, (g) edible sprouts, and (h) the details of sprout. **(C)** Headed bróquil type: (i) plant in the field, (j) sprouts in the commercial stage, (k) edible sprouts, and (l) detail of sprout.

The plant material was provided by the Spanish Vegetable Genebank of the Agrifood Research and Technology Center of Zaragoza (BGHZ-CITA). Seeds of 13 landraces of bróquil (*B. oleracea*, var. *italica*) were used for this study: six headed bróquil accessions (HB) and seven green bróquil accessions (GB) (Figure 1 and Table 1). The commercial broccoli variety “Parthenon” was also included in the assay as a control.

**Table 1:** Identification and origin of studied bróquil accessions (*B. oleracea* var. *italica*) from the Spanish Vegetable Genebank of Zaragoza (BGHZ-CITA).

**Experiment code**	**Genebank code**	**National inventory code**	**Bróquil type**	**Origin: Locality, province, country**
HB1	BGHZ7610	In progress	Headed bróquil	Barbastro, Huesca, Spain
HB2	BGHZ7154	NC113789	Headed bróquil	Huesca, Spain
HB3	BGHZ2636	NC075031	Headed bróquil	Huesca, Spain
HB4	BGHZ2637	NC075032	Headed bróquil	Huesca, Spain
HB5	BGHZ6685	NC111436	Headed bróquil	Binéfar, Huesca, Spain
HB6	BGHZ6687	NC104640	Headed bróquil	Loporzano, Huesca, Spain
GB1	BGHZ7155	NC113790	Green bróquil	Huesca, Spain
GB2	BGHZ6972	NC104641	Green bróquil	Loporzano, Huesca, Spain
GB3	BGHZ2638	NC075033	Green bróquil	Huesca, Spain
GB4	BGHZ3021	NC074979	Green bróquil	Barbastro, Huesca, Spain
GB5	BGHZ4057	NC082528	Green bróquil	Calatayud, Zaragoza, Spain
GB6	BGHZ6686	NC111437	Green bróquil	Azlor, Huesca, Spain
GB7	BGHZ6688	NC111438	Green bróquil	Barbastro, Huesca, Spain

The assay was carried out in the experimental fields of CPIFP Montearagón (42.10522, −0.37988) Huesca, Spain, during the fall-winter of 2019–2020 using a randomized complete block design with three replications. Each replication consisted of 14 plants.

The seeds of 13 bróquil and commercial broccoli were sown in foam trays filled with a mixture of peat moss and vermiculite (1:1 volume). Soil preparation, planting, and other agronomic practices were carried out uniformly following the standard bróquil growth practices. The seedlings were transplanted at the third true leaf stage on the 12th of September with a 60 cm distance between the plants and a row-to-row distance of 60 cm, equivalent to a crop density of 2.77 plants m^−2^ in outdoor cultivation. Lines of the drip irrigation system were implanted following the plant row. No mineral fertilizers were applied. Previous crops were legumes. No signs of aphids, lepidoptera, fungi, viruses, other plant insect attacks, or infection were detected, and thus, no phytosanitary treatment was necessary.

### 2.2. Preparation of plant samples for chemical analysis

Three plants per replication were harvested at the commercial maturity stage and transported immediately to the laboratory. Broccoli was ready to harvest when the heads are well formed and green but still in the compact bud in the same way as bróquil but yellowish. Before harvesting, the edible parts were separated, washed, chopped, and the moisture content was determined using an oven at 105°C for 24 h. For each field replication, 100 g was prepared and frozen for later lyophilization. The freeze-dried samples were milled in a ball mill. The samples were stored in airtight plastic bags in a freezer at −18°C until analysis. The analyses were performed in triplicate, and the results were expressed based on dry weight for each sample.

### 2.3. Determination of bioactive compounds

The extract from freeze-dried bróquil samples was prepared by following the procedure described by Mawlong et al. (17) with slight modifications. To 0.25 g of the freeze-dried bróquil sample, 5 ml of 80% methanol was added and this homogenate was centrifuged at 3,000 rpm for 10 min after keeping overnight in darkness at 20°C. The methanol extract was filtered (0.45 μm) and kept at −18°C until analysis for the determination of total phenolic content (TPC), total flavonoid content (TFC), and total glucosinolate content (TGLC). All determinations were carried out spectrophotometrically using a UV-vis spectrophotometer (UV2 spectrometer, Unicam).

For TPC determination, the Folin–Ciocalteu method was used with slight modifications (18). To 0.5 ml of the methanolic extract, 0.5 ml of Folin–Ciocalteu reagent and 8 ml of distilled water were added. After 5 min in an ultrasonic bath, 1 ml of 20% sodium carbonate (Na_2_CO_3_) was added. An intense blue color developed after 30 min in the dark at ambient temperature and its absorbance was measured at 760 nm. Gallic acid was used as a standard for calibration at different concentrations (2–20 mg L^−1^). The results were expressed as mg of gallic acid equivalents per gram of the dry weight sample (mg GAE g^−1^ dw).

For TFC determination, the aluminum chloride method was used (19). To 0.5 ml of the methanolic extract, 0.5 ml of 80% methanol, 4 ml of distilled water, and 0.3 ml of 5% sodium nitrite (NaNO_3_) were added. After 5 min of incubation, 0.3 ml of 10% aluminum chloride (AlCl_3_) was added. Before 1 min, 2 ml of 1 M sodium hydroxide (NaOH) was added. Finally, the volume was made up to 10 ml with distilled water and homogenized. An orange-yellowish color was developed and the absorbance was measured at 510 nm. Quercetin was used as a standard for calibration at different concentrations (4–30 mg L^−1^). The results were expressed as mg of the quercetin equivalents per gram of the dry weight sample (mg QE g^−1^ dw).

For TGLC determination, the tetrachloropalladate method was used (17). To 0.5 ml of the methanolic extract, 0.3 ml of distilled water and 3 ml of 2 mM sodium tetrachloropalladate (Na_2_PdC_l4_) were added. After incubation at 20°C for 1 h, absorbance was measured at 425 nm. Sinigrin was used as a standard for calibration at different concentrations (10–90 mg L^−1^). The results were expressed as mg of the sinigrin equivalents per gram of the dry weight sample (mg SnE g^−1^ dw).

The total content of vitamin C [the sum of ascorbic acid (AA) and dehydroascorbic acid (DHAA)] was determined by the 2,6-dichloroindophenol (DCIP) spectrophotometric method (20) and the modified procedure for extraction applied by Medina et al. (21). To 0.25 g of the freeze-dried sample, 2.5 ml of the extractant reactive (3% (m/v) metaphosphoric acid and 8% (v/v) acetic acid) was added and homogenized in a vortex for 1 min. This homogenate was centrifuged at 3,000 rpm for 10 min after keeping it at 4°C for 15 min. The supernatant was filtered (0.45 μm PTFE) and reserved for analysis. To 0.2 ml of the extract, 0.2 ml of 1.5 mM 1,4-dithiothreitol (DTT) was added to reduce all DHAA to AA. After incubation for 30 min at ambient temperature, 0.2 ml of 0.4 M H_2_SO_4_ was added. To 0.1 ml of this mix, 0.9 ml of 15% (m/v) DCPIP was added and the absorbance was measured immediately after mixing for 15 s at 515 nm. Ascorbic acid was used as a standard for calibration at different concentrations (10–140 mg L^−1^). The results were expressed in mg ascorbic acid per gram of the dry weight sample (mg AA g^−1^ dw).

### 2.4. Antioxidant activity

Samples were prepared as described previously for determining the activities of the bioactive compounds. For the determination of antioxidant activity (AA), the DPPH (2,2-diphenyl-1-picrylhydrazyl) radical method was used (22). This method was optimized and adapted for bróquil in a previous study (Data not shown). Briefly, 0.2 ml of the methanolic extract was mixed with 1 ml of DPPH (0.3 g L^−1^) and the absorbance was measured at 510 nm after keeping it at 10 min at ambient temperature. A blank control was prepared and measured with 0.2 ml of 80% methanol and 1 ml of DPPH. The lower absorbance of the reaction mixture after a 10-min reaction indicated higher free radical scavenging activity. The results were expressed as percentage inhibition (%I) using the following equation: %I = ((Ac - As) / Ac) × 100.

where *Ac* is the absorbance of the control (blank) and *As* is the absorbance in the presence of the bróquil methanolic extract.

Besides, AA is expressed as an activity equivalent of Trolox (mg Trolox g^−1^ of dry weight sample), (mg TE g^−1^ dw), according to previous studies using the following equation: *y* = 0.0164*x* – 0.4719 (*R*^2^ = 0.9924), where *x* is the %I.

### 2.5. Determination of mineral composition

The mineral composition was determined by the dry ash method (23). For mineral determination, 1 g of the freeze-dried sample was calcined in a muffle at an initial temperature of 200°C for 1 h. Subsequently, it was kept at 480°C overnight. The ashes were dissolved in a hot plate with a mixture of 4 ml of nitric acid (65%) and 10 ml of distilled water. The mineral solution was filtered and made up to 25 ml with distilled water. This solution was used for elemental analysis. The mineral content (ashes) was determined by weight difference. The determination of macro- and microelements (Ca, Mg, Na, K, Fe, Mn, and Zn) was performed by atomic absorption/emission spectroscopy (Varian SpectrAA-110 spectrophotometer). The calibration curves were made by diluting the standards to the specific concentrations for each element. The results were expressed as mg element per gram of the dry weight sample.

For the determination of P and S, spectrophotometric methods based on the standard methods for water analysis (24) were used and adapted to bróquil sample dissolution. The concentration of S was determined by the barium sulfate turbidimetric procedure. To 0.4 ml of the solution sample, 1 ml of 2 M HCl and 1 ml of reagent (0.5 M BaCl_2_ and 1.5% agar-agar) were added and made up to a volume of 10 ml with distilled water and shaken. After 10 min, the absorbance was measured at 420 nm. Sodium sulfate (Na_2_SO_4_) was used as a standard for calibration at different concentrations of sulfate (5–40 mg L^−1^). The results were expressed in mg sulfur per gram of the dry weight sample. The concentration of *P* was determined by the colorimetric procedure using acid ammonium molybdate. To 0.03 ml of the solution sample, 3 ml of distilled water was added and the pH was adjusted to 3–10 with 1 M NaOH. Then, 0.2 ml of ascorbic acid and 0.4 ml of a mixed solution (0.04 M ammonium molybdate, 0.02 M potassium antimony tartrate, and 3 M sulfuric acid) were added and made up to 10 ml with distilled water and shaken. After 10 min, the absorbance of the molybdenum blue complex was measured at 880 nm. Potassium acid phosphate (K_2_HPO_4_) was used as a standard for calibration at different concentrations of phosphate (0.1–1.0 mg L^−1^). The results were expressed in mg phosphorus per gram of the dry weight sample.

### 2.6. Statistical analysis

The results were analyzed from the mean of determinations for duplicate samples prepared for each genotype. Data were expressed as the mean ± standard deviation (SD). The mean was compared using the one-way analysis of variance (ANOVA) followed by a *post-hoc* Tukey-b test to construct homogeneous groups. The differences between individual means were deemed to be significant at a *p*-value of < 0.05. All analyses were performed using the SPSS statistical package (SPSS for Windows, version 16.0).

## 3. Results

### 3.1. Phytochemical compounds

The phytochemical analysis of bróquil included total phenolic, flavonoid, and glucosinolate contents and vitamin C. The results presented were obtained from the analysis of dried and subsequently freeze-dried plant material. The moisture range was 86.3–91.2% for headed bróquil, 83.0–89.8% for green bróquil, and 87.5% for broccoli.

### 3.2. Total phenolic content (TPC)

**Figure 2:**
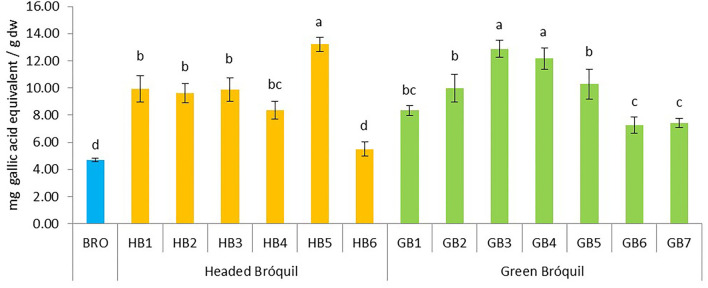
Total phenolic content of 13 bróquil accessions (six headed bróquil: HB1 to HB6 and seven green bróquil: GB1 to GB7) and broccoli control (BRO). Each bar represents the average data and standard deviation (±SD) of phenol content per accession expressed as mg of gallic acid equivalent per gram of dry weight (dw). Different letters show statistically significant differences between the groups (*p* < 0.05).

The total phenol content of bróquil ranged from 5.49 (HB6) to 13.21 mg GAE g^−1^ dw (HB5). Minimum values (4.69 mg g^−1^) were obtained for broccoli. The plant material showed significant differences between accessions (Figure 2). Data could be grouped into four groups based on variance analysis. Three genotypes, HB5, GB3, and GB4 statistically have the same contents but show significant differences with respect to all the other samples. Seven accessions HB1, HB2, HB3, HB4, GB1, GB2, and GB5 did not show significant differences among them. Bróquil TPC was higher than that of broccoli, although sample HB6 and broccoli control did not show significant differences. The ANOVA analysis comparing green vs. headed bróquil did not detect significant differences, probably due to the heterogeneity of both types.

### 3.3. Total flavonoid content (TFC)

**Figure 3:**
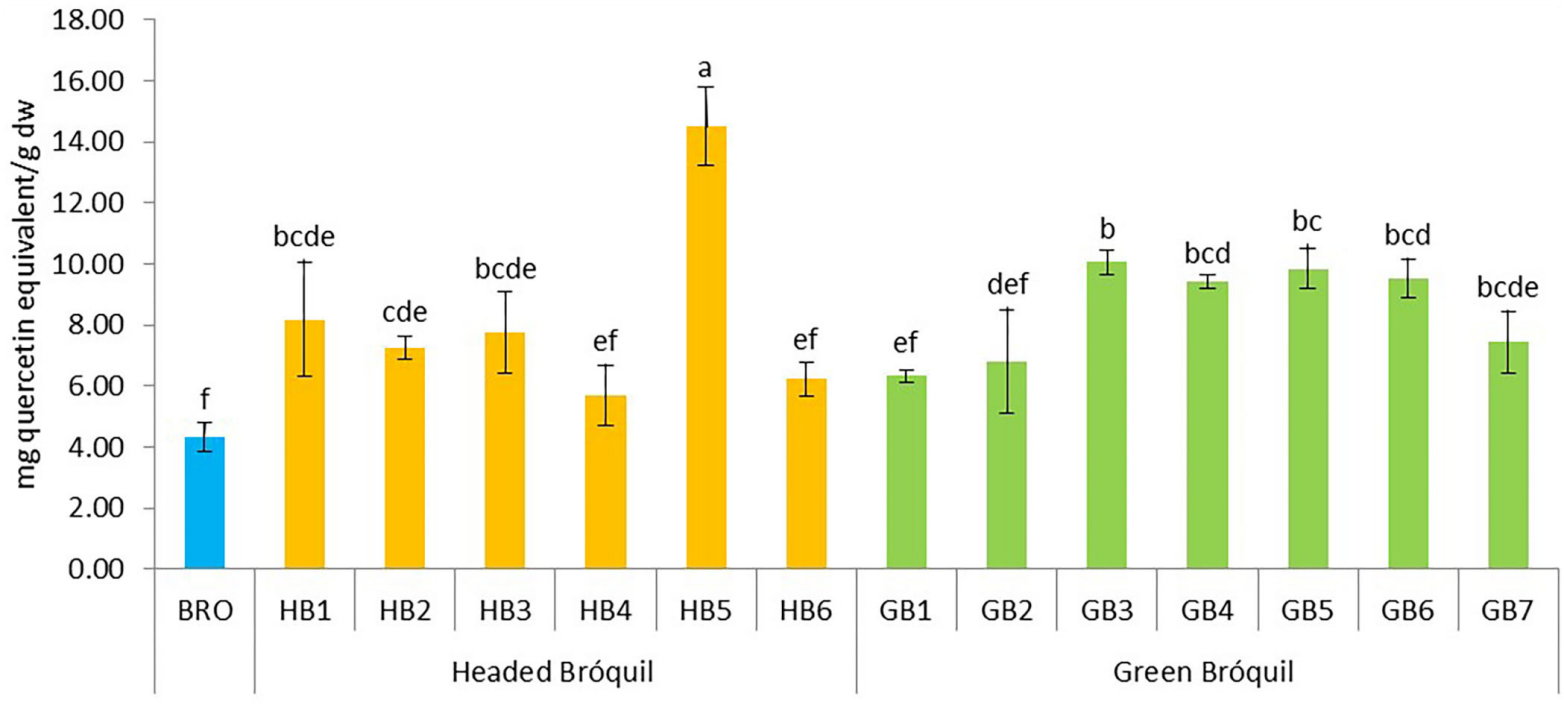
Total flavonoid content of 13 bróquil accessions (six headed bróquil: HB1 to HB6 and seven green bróquil: GB1 to GB7) and broccoli control (BRO). Each bar represents the average data and standard deviation (±SD) of total flavonoid content per accession expressed as mg quercetin equivalent per gram of dry weight (dw). Different letters show statistically significant differences between the accessions (*p* < 0.05).

The total flavonoid content varied from 4.32 mg QE g^−1^ dw in broccoli to 14.50 mg QE g^−1^ dw in HB5 (Figure 3). Significant differences were observed among accessions and six homogeneous groups were identified, evidencing the variability of the accessions for this bioactive compound. All bróquil accessions showed higher TFC than the broccoli control. Among bróquil accessions, HB5 presented a higher content, significantly different from the rest of the accessions. No clear differences were observed between headed and green bróquil.

### 3.4. Total glucosinolate content (TGLC)

**Figure 4:**
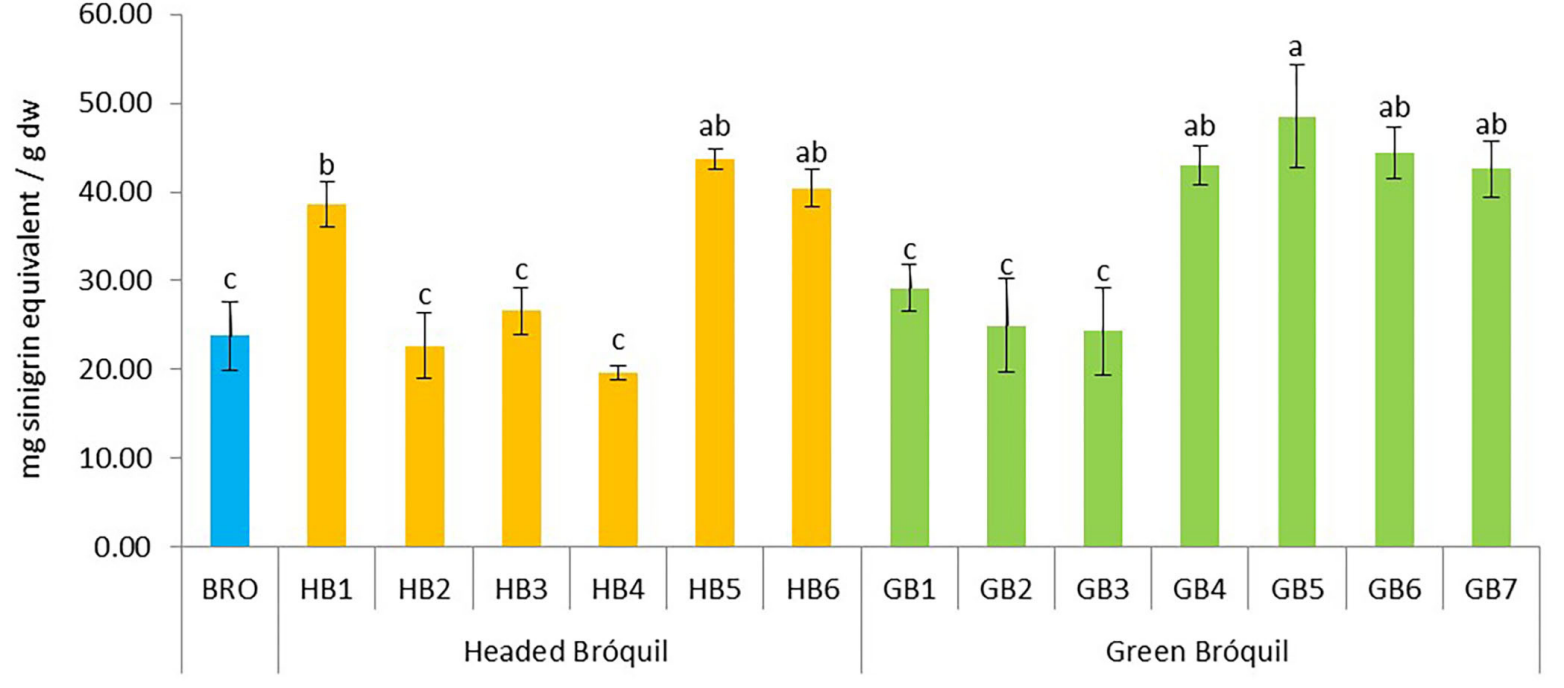
Total glucosinolate content of 13 bróquil accessions (six headed bróquil: HB1 to HB6 and seven green bróquil: GB1 to GB7) and broccoli control (BRO). Each bar represents the average data and standard deviation (±SD) of the total glucosinolate content per accession expressed as mg of sinigrin equivalent per gram of dry weight. Different letters show statistically significant differences between the groups (*p* < 0.05).

The total glucosinolate content ranged from 19.62 (HB4) to 48.51 mg SnE g^−1^ dw (GB5) and significant differences were detected between all accessions (Figure 4). Three homogeneous subsets were established. HB5, HB6, GB4, GB5, GB6, and GB7 showed high glucosinolate content, and no significant differences between them were detected. In the same way, HB2, HB3, HB4, GB1, GB2, GB3, and BRO showed the lowest values, and no significant differences were detected. In general, bróquil glucosinolate contents were similar to or higher than broccoli.

### 3.5. Vitamin C

**Figure 5:**
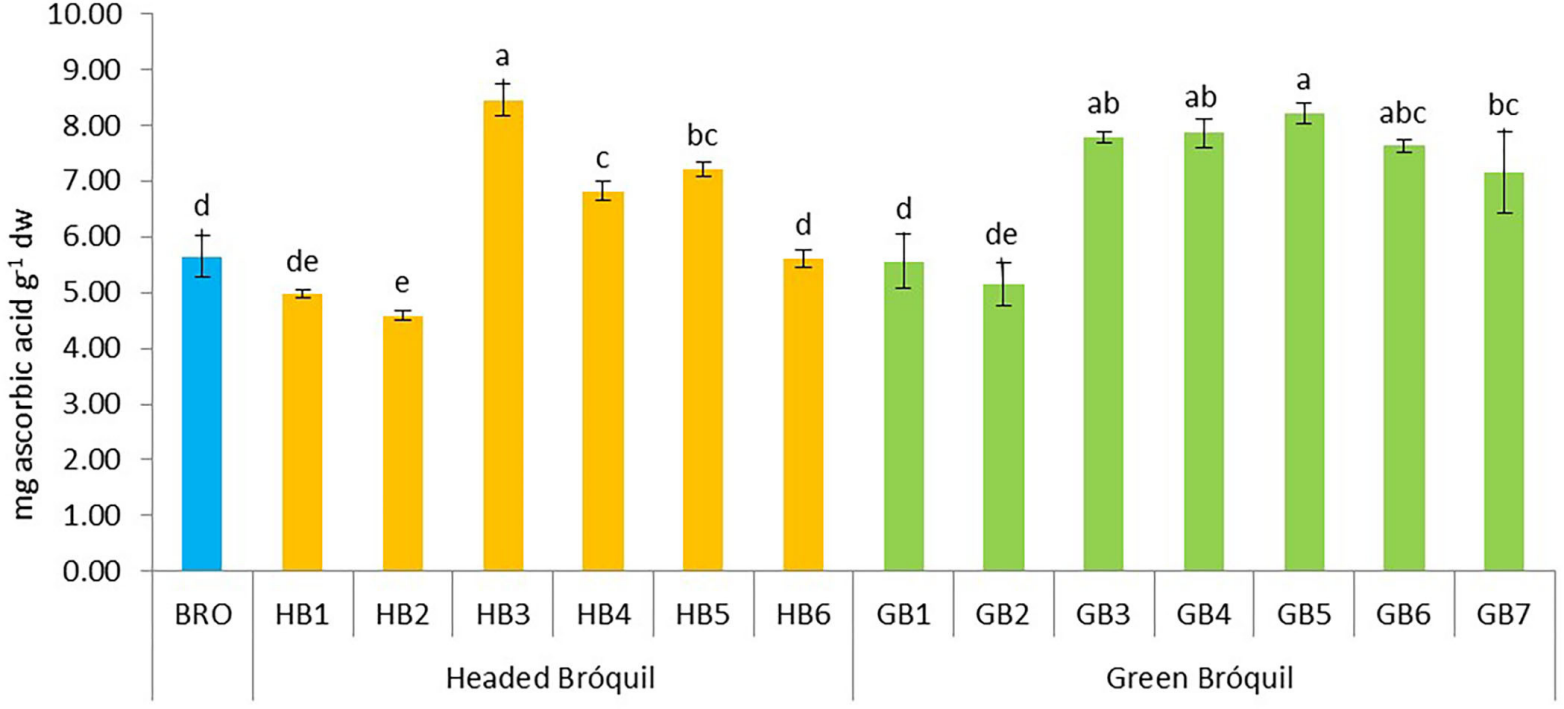
Vitamin C content of 13 bróquil accessions (six headed bróquil: HB1 to HB6 and seven green bróquil: GB1 to GB7) and broccoli control (BRO). Each bar represents the average data and standard deviation (±SD) of vitamin C content per accession expressed as mg of ascorbic acid per gram of dry weight. Different letters show statistically significant differences between the accessions (*p* < 0.05).

Vitamin C content ranged from 4.59 (HB2) to 8.45 mg AA g^−1^ (HB3). Significant differences were detected between accessions (Figure 5). Four accessions of green bróquil (GB3, GB4, GB5, and GB6) followed by headed bróquil HB3 had the highest vitamin C content without significant differences between them. Bróquil vitamin C content is similar to or higher than that of broccoli exception for HB2 which showed the lowest value.

### 3.6. Antioxidant activity

**Figure 6:**
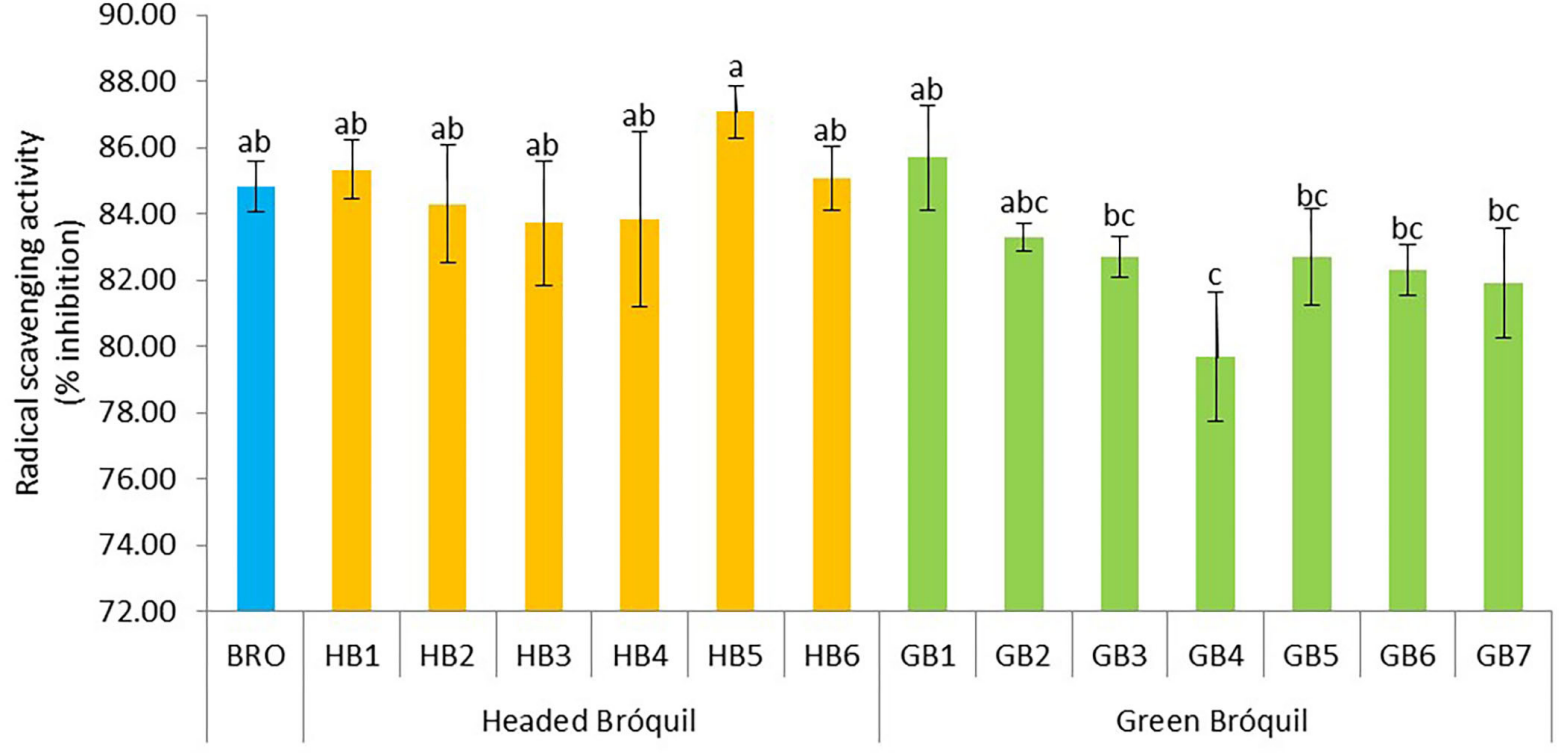
Antioxidant activity of 13 bróquil accessions (six headed bróquil: HB1 to HB6 and seven green bróquil: GB1 to GB7) and broccoli control (BRO). Each bar represents the average data and standard deviation (±SD) of antioxidant activity per accession expressed as radical scavenging activity (% inhibition). Different letters show statistically significant differences between the accessions (*p* < 0.05).

Low variability was detected among bróquil accessions in terms of antioxidant activity (Figure 6). Data ranged from 79.77 (GB4) to 87.07 %I (HB5). These data correspond to 0.83–0.96 mg TE g^−1^ dw. No significant differences were detected between headed bróquil accessions and green bróquil ones. Notably, the high standard deviation among the accessions evidences the heterogeneity of these landraces regarding the antioxidant activity. Except for GB4, bróquil accessions did not show statistically significant differences regarding antioxidant activity when compared to broccoli.

### 3.7. Mineral compounds

The present study revealed that the average ash content in bróquil samples vary from 7.02 to 9.24 g per 100 g of dry matter in samples GB4 and HB3, respectively, while in broccoli, the ash content was 7.14 g per 100 g of dry matter. All macronutrients and microelements showed significant differences among the bróquil accessions and broccoli control (Tables 2, 3).

**Table 2:** Contents of ash (g 100 g^−1^ dw) and macroelements (mg g^−1^ dw) in headed bróquil (HB), green bróquil (GB), and broccoli control (BRO) accessions.

		**Ash (g/100 g dw)**	**Macroelements (mg/g dw)**					
			**Phosphorus**	**Potassium**	**Sulfur**	**Magnesium**	**Calcium**	**Sodium**
Headed bróquil (HB)	*HB1*	7.72 ± 0.45^cdef^	5.12 ± 0.21^bcd^	25.22 ± 2.71^de^	4.94 ± 0.25^bc^	1.37 ± 0.02^de^	6.91 ± 0.50^c^	0.30 ± 0.03^bcde^
	*HB2*	7.37 ± 0.36^ef^	6.18 ± 0.43^abc^	32.78 ± 0.93^a^	3.56 ± 0.30^fg^	1.40 ± 0.08^de^	4.56 ± 0.15^ef^	0.30 ± 0.03^bcd^
	*HB3*	9.24 ± 0.25^a^	6.33 ± 0.42^abc^	32.10 ± 1.08^ab^	4.77 ± 0.31^bcd^	1.71 ± 0.12^ab^	7.63 ± 0.68^abc^	0.40 ± 0.01^bc^
	*HB4*	8.86 ± 0.34^ab^	7.48 ± 0.63^a^	33.62 ± 2.63^a^	4.07 ± 0.16^def^	1.69 ± 0.20^abc^	6.10 ± 0.49^cde^	0.40 ± 0.05^b^
	*HB5*	7.94 ± 0.08^bcdef^	4.90 ± 0.18^cd^	29.16 ± 1.96^bc^	5.12 ± 0.05^b^	1.42 ± 0.06^cde^	6.62 ± 1.70^cd^	0.30 ± 0.01^bcde^
	*HB6*	7.82 ± 0.17^bcdef^	6.62 ± 0.54^ab^	26.33 ± 1.15^cde^	3.27 ± 0.11^g^	1.37 ± 0.14^de^	4.07 ± 0.54^f^	0.30 ± 0.04^bcd^
Green bróquil (GB)	*GB1*	8.73 ± 0.44^abc^	6.46 ± 0.75^ab^	33.60 ± 0.78^a^	3.92 ± 0.42^efg^	1.80 ± 0.03^a^	6.74 ± 0.64^cd^	0.30 ± 0.01^bcd^
	*GB2*	8.26 ± 0.02^abcde^	6.89 ± 0.83^a^	29.18 ± 1.64^bc^	3.19 ± 0.29^g^	1.65 ± 0.05^abcd^	5.27 ± 0.49^def^	0.30 ± 0.06^bcde^
	*GB3*	8.25 ± 0.77^abcde^	6.41 ± 0.50^abc^	24.69 ± 0.68^e^	5.25 ± 0.23^b^	1.48 ± 0.07^bcde^	8.54 ± 0.83^ab^	0.30 ± 0.07^bcd^
	*GB4*	7.02 ± 0.76^f^	4.59 ± 0.51^d^	24.15 ± 1.03^e^	4.52 ± 0.41^bcde^	1.40 ± 0.20^de^	6.48 ± 0.50^cd^	0.20 ± 0.03^de^
	*GB5*	7.62 ± 0.29^def^	5.37 ± 0.11^bcd^	26.34 ± 0.66^cde^	4.62 ± 0.23^bcde^	1.29 ± 0.08^e^	7.27 ± 0.52^bc^	0.20 ± 0.02^e^
	*GB6*	8.56 ± 0.24^abcd^	5.24 ± 0.61^bcd^	28.76 ± 0.67^bcd^	6.94 ± 0.51^a^	1.25 ± 0.09^e^	9.01 ± 0.47^a^	0.30 ± 0.05^cde^
	*GB7*	7.29 ± 0.14^ef^	5.37 ± 0.68^bcd^	22.66 ± 1.32^e^	3.50 ± 0.26^fg^	1.28 ± 0.12^e^	6.03 ± 0.71^cde^	0.20 ± 0.03^e^
Broccoli control	*BRO*	7.14 ± 0.27^ef^	5.98 ± 0.41^abcd^	25.64 ± 0.83^cde^	4.21 ± 0.29^cdef^	1.89 ± 0.02^a^	4.91 ± 0.46^ef^	1.10 ± 0.06^a^

Values are presented as mean value ± SD (n = 3). Different letters in the same column indicate a significant difference at p < 0.05.

**Table 3:** Contents of microelements (mg g^−1^dw) in headed bróquil (HB), green bróquil (GB), and broccoli control (BRO) accessions.

		**Microelements (mg/g dw)**		
		**Iron**	**Manganese**	**Zinc**
Headed bróquil (HB)	*HB1*	0.132 ± 0.008^ab^	0.025 ± 0.002^bcd^	0.022 ± 0.001^f^
	*HB2*	0.135 ± 0.005^a^	0.019 ± 0.002^d^	0.036 ± 0.007^de^
	*HB3*	0.126 ± 0.001^ab^	0.029 ± 0.002^ab^	0.051 ± 0.004^abc^
	*HB4*	0.133 ± 0.007^ab^	0.027 ± 0.002^abc^	0.061 ± 0.005^a^
	*HB5*	0.122 ± 0.014^ab^	0.026 ± 0.003^bc^	0.045 ± 0.007^bcd^
	*HB6*	0.125 ± 0.009^ab^	0.023 ± 0.004^bcd^	0.054 ± 0.006^ab^
Green bróquil (GB)	*GB1*	0.122 ± 0.001^ab^	0.020 ± 0.002^cd^	0.045 ± 0.004^bcd^
	*GB2*	0.127 ± 0.015^ab^	0.022 ± 0.003^bcd^	0.050 ± 0.007^abc^
	*GB3*	0.135 ± 0.010^a^	0.033 ± 0.004^a^	0.050 ± 0.005^abc^
	*GB4*	0.125 ± 0.004^ab^	0.029 ± 0.002^ab^	0.037 ± 0.002^de^
	*GB5*	0.126 ± 0.003^ab^	0.029 ± 0.002^ab^	0.040 ± 0.005^cde^
	*GB6*	0.106 ± 0.009^bc^	0.029 ± 0.003^ab^	0.031 ± 0.004^ef^
	*GB7*	0.119 ± 0.018^ab^	0.024 ± 0.003^bcd^	0.029 ± 0.003^ef^
Broccoli control	*BRO*	0.088 ± 0.006^c^	0.023 ± 0.002^bcd^	0.058 ± 0.002^a^

Values are presented as mean ± SD (n = 3). Different letters in the same column indicate a significant difference at p < 0.05.

### 3.8. Macroelements

The main ash component was potassium ranging from 22.66 to 33.62 mg g^−1^ for GB7 and HB4, respectively. The broccoli control presented an intermediate value of 25.64 mg g^−1^ and was included in the homogeneous group with a lower potassium content. In contrast, bróquil accessions HB2, HB3, HB4, and GB1 have higher potassium values. The following macroelements were phosphorus, calcium, and sulfur, with average values ranging from 6 mg g^−1^ in all cases. Magnesium and sodium were the macroelements with the lowest contents, with values below 2 mg g^−1^. Green and headed bróquil did not show differences according to their macroelement content. The broccoli variety, used as the control, had high values of magnesium (1.89 mg g^−1^), thus establishing a homogeneous group with HB3 and GB1 and highlighting its high sodium content of 1.10 mg g^−1^, since the bróquil accessions presented values between 0.20 and 0.40 mg g^−1^.

### 3.9. Microelements

Broccoli's iron content of 0.088 mg g^−1^ was significantly lower than the values obtained for bróquil accessions, except for GB6. The iron content of bróquil accessions ranged from 0.135 (GB3 and HB2) to 0.106 mg g^−1^ (GB6). The maximum and minimum contents of manganese were obtained for bróquil accessions, especially GB3 (0.033 mg g^−1^) and HB2 (0.019 mg g^−1^), while broccoli showed an intermediate value of 0.023 mg g^−1^. The higher values for zinc were obtained from the broccoli (0.058 mg g^−1^) and the bróquil accessions HB3, HB4, HB6, GB2, and GB3. The minimum zinc content obtained was 0.022 mg g^−1^ from the HB1 accession.

## 4. Discussion

This study shows the analytical data of the bioactive compounds, antioxidant activities, and mineral compositions of a collection of 13 bróquil accessions, a *Brassica* landrace maintained in a small area of the Spanish Aragón region. The work constitutes an approach to the bioactive properties of this type of Brassicas performed for the first time since no previous bibliographical references exist. Consequently, the potential beneficial effects of bróquil may only contrast the *B. oleracea* typologies. *Brassica* species and, in particular, broccoli are reputed for their nutritional value. They are a source of phytochemicals (e.g., glucosinolates and phenolic compounds) and diverse nutrients (e.g., minerals and vitamins) (15, 25, 26). In this study, these parameters have been studied in bróquil and the results revealed a great variability among and within accessions. As it is difficult to compare our bróquil research with other studies, the control broccoli was also included in the experimental design. It is important to highlight that, in this study, all the plant material was grown under the same conditions to minimize the influence of agronomical and experimental conditions, such as fertilization or climatology. Similarly, we can observe the influence of the genotype on the obtained results.

According to our results, phenolic compounds are the important bioactive compounds in bróquil. On average, in our experimental conditions, the TPC content of all bróquil landraces was 9.6 mg GAE g^−1^ dw which is higher than broccoli (4.7 mg GAE g^−1^ dw). These results range from 3.7 to 12.1, 7.5 to 4.6, and 6.4 mg GAE g^−1^ dw for commercial broccoli, Italian broccoli landraces, and broccoli as reported by Koh et al. (27), Nicoletto et al. (16), and Tabart et al. (28), respectively. Although the aforementioned limitations must be considered to compare these results, the quality and quantity of phenolic compounds in the plants vary significantly due to different factors, such as, genetic determinants (29), and environmental conditions, like fertilization (30).

The most widespread and diverse group of polyphenols in *Brassica* species are the flavonoids (31). Flavonoids are a class of naturally occurring secondary metabolites, which are synthesized in plants through the phenylpropanoid pathway. Flavonoids are widely distributed in plants and fulfill many important functions for plant growth and development (32, 33). Besides, flavonoids are gaining increasing interest due to their diverse health benefits to humans. They have been shown to be strong antioxidant and anti-inflammatory agents in the human diet (34, 35), and consumption of high-flavonoid foods has been associated with reduced risk of several chronic diseases (36). Among all the vegetables, broccoli is the main flavonoid source in our diet (37). Our results showed, on average, that the TFC of bróquil (8.3 mg QE g^−1^ dw) was higher than that obtained for broccoli (4.3 mg QE g^−1^ dw), which suggests a higher nutritional as well as nutraceutical value of bróquil cultivars. Naguid et al. (30) found higher values of 14.3 and 14.2 mg QE g^−1^ dw for Calabrese and Southern Star broccoli cultivars respectively. Such fluctuations might be due to several factors, including genotype, developmental stages, agronomic environment, and postharvest conditions (27, 29). Among these factors, fertilization has been reported as a highly significant factor influencing TFC accumulation (30). Our lower values may be due to the lack of fertilization during the assay.

Glucosinolates, the water-soluble secondary plant metabolites, are the characteristic compounds in Brassica vegetables (38). The consumption of Brassica vegetables has been reported to be effective against cancer, preventing cardiovascular diseases or lowering the risks of various chronic degenerative diseases, which are mediated through glucosinolates, a source of bioactive isothiocyanates for human nutrition and health (39, 40). Under our experimental conditions, on average, the levels of glucosinolates were higher in headed bróquil than in green accessions and also higher than the commercial broccoli grown under the same agronomic conditions as the control. Specifically, our results showed the TGLC average values of 23.7 mg SnE g^−1^ dw for broccoli and 34.2 mg SnE g^−1^ dw for bróquil (31.9 for HB and 36.7 for GB). Six bróquil accessions showed similar TGLC values while seven bróquil accessions, including both headed and green types, showed significantly higher values than broccoli. Bhandari et al. (22) found glucosinolate values that ranged from 10.5 to 13.8 mg SnE g^−1^ dw in a group of six broccoli genotypes at the commercial stage of maturation, which were lower than our broccoli control. Due to the high values, the obtained results identify promising bróquil accessions for TGLC, forming a homogeneous group that ranged from 38.6 to 48.5 mg SnE g^−1^ dw.

Vitamin C, an antioxidant, is an essential nutrient for the human body. Brassica foods have been described as a dietary source of this vitamin, which may work synergistically with the wealth of bioactive compounds present in these foods (41). The content of vitamin C was 6.6 mg AA g^−1^ dw on average in bróquil and 5.6 mg AA g^−1^ dw in broccoli. The obtained values are higher than 2.5–4, and 1.18 mg AA g^−1^ dw previously described for broccoli by Nicoletto et al. (16) and Tabart et al. (28). The differences may be due to our results being calculated as the total vitamin C content, considering ascorbic acid (AA) and dehydroascorbic acid (DHAA), while Tabart et al. (28) only determined ascorbic acid. Another reason may be due to the spectroscopic method of analysis, while Nicoletto et al. (16) used the spectrofluorometric method. Although some bróquil accessions showed similar values, others showed significantly higher values than broccoli, reinforcing the nutritional interest of this brassica.

Over the past decades, literature has noted the interest in *Brassicaceae* vegetable production thanks to the scientific evidence that showed the health benefits of their antioxidant compounds and related antioxidant properties (42). The antioxidant capacity is contributed by many biologically active biomolecules, such as vitamin C, phenolic compounds, glucosinolates, carotenoids, and others in the plants (43) as the antioxidant capacity is a composite result of complex factors (37). Bróquil with an average of 83.7%I showed similar values to broccoli (84.9 %I), equivalent to 0.90 and 0.92 mg of TE g^−1^ dw, respectively. Zhang and Hamauzu (44) described in broccoli 61.7 %I and Rivera-Martín et al. (45) found a content of 0.71 mg of TE g^−1^ dw, with both data lower or similar to bróquil results obtained using the same method. On the other side, as previously stated, broccoli has been described as a source of bioactive compounds, which possess antioxidant effects. However, these attributes can be also applied to bróquil. Nevertheless, the high variability observed in the bioactive compounds determined in bróquil is not reflected in the variability of antioxidant activity. According to Cartea et al. (31), phenolic compounds exhibit higher antioxidant activity compared to vitamin C and carotenoids. However, the higher content of phenolic compounds did not always demonstrate a higher antioxidant capacity, possibly as a result of the antagonistic effect between the different phenolic classes. Therefore, the high values obtained for antioxidant activity may be influenced by other key contributors like carotenoids, chlorophylls, or other vitamins not quantified in this study.

During the past decade, consumption of *B. oleracea* edible crops has been highly recommended, based on the presence of secondary plant metabolites with health protective effects. Apart from these compounds, Brassicas might provide high levels of minerals in comparison with other groups of vegetables (46). However, they are likely to be affected by the cultivar, environment, and type of edible part (47). Our results reinforce this statement, as bróquil has a significant source of minerals. As shown in Tables 2, 3, bróquil and broccoli contain macronutrients and micronutrients that are crucial for human wellbeing and health. All macroelements and microelements seem to be dependent on the bróquil genotype and, compared to broccoli, showed higher or similar values.

The variability reported in this study clearly shows that the contents of macronutrients and micronutrients in bróquil accessions depend on the genotype. The content of macronutrients in bróquil landraces follows the sequence: K > Ca > P > S > Mg > Na. The content of micronutrients is arranged as follows: Fe > Mn > Zn. Among the macronutrients, the content of K was the highest, followed by those of Ca, P, and S whose values were relatively lower compared to K. The level of Mg was relatively lower compared to the others. We can also highlight the lower amount of Na obtained for all bróquil accessions. A normal amount of K in a typical diet of healthy people is about 2–5.9 g^−1^ days and the minimum daily requirement is estimated to be 782 mg (48). Transforming results obtained in this study, on average, a portion of 100 g of fresh bróquil implies 350 mg of K, which means that bróquil could be considered as a potassium-rich food. In this study, the accessions with the highest K levels were HB4 and GB1, which accumulated up to 33.6 mg g^−1^ dw (418.5 mg K 100 g^−1^ fw).

On the contrary, calcium is one of the most important minerals. It is vital for the skeleton and the good functioning of nerves and muscle tissue. People at different life stages need different amounts of calcium but the desirable calcium intake is stipulated between 1.2 and 1.5 g per day for adults (48). Bróquil landraces showed on average 81 mg of 100 g^−1^ fresh weight (about 7% of total intake). The bioavailability of calcium (Ca) in Brassicas is compared to milk (47), reinforcing the importance of this vegetable as a calcium source. Studies showed that broccoli is an important alternative source of Ca in the segments of the population that consume limited amounts of dairy products (49). According to our results, this can also be applied to bróquil.

Cruciferous are characterized as one of the main sources of sulfur in the vegetable kingdom. While there is no recommended daily allowance for sulfur and considering that animal-based proteins were the primary sources of daily intakes, consumption of bróquil can contribute to getting plenty of sulfur, especially in vegetarian diets. Our results showed lower values than those reported previously by Rosa et al. (47)—between 8.9 and 18.6 mg ^−1^ but higher than that obtained by Xiao et al. (50) of 2.6–4.3 mg g^−1^.

Regarding micronutrients, iron was the most abundant detected in our study. Iron was higher in all bróquil accessions than in broccoli, except for GB6. Iron is a vital component of proteins involved in oxygen transport and metabolism and an essential cofactor in the synthesis of neurotransmitters. Approximately 15% of the body's iron is stored for future needs and mobilized when dietary intake is inadequate (51). Although the iron requirement depends on the age and sex of the individual, it is about 1.5–2.2 mg/day. Our results showed, on average, a content of 2.7 mg 100 g^−1^ fw. Although this data suggests that bróquil could be considered a high source of iron, we have to take into account that the absorption of iron in vegetables is very low, from 1.0 to 1.5% (48).

In summary, we studied for the first time the available genetic resources for the two types of bróquil (green and headed) which comprises all the diversity for this Spanish local edible brassica. The results describe their mineral content, bioactive compounds, and antioxidant activity. No differences were detected between green and headed bróquil; however, a great variability within each typology was observed, due to the lack of a formal breeding selection. The results show promising contents of total phenolic compounds, flavonoids, glucosinolates, and vitamin C. The antioxidant activity value of bróquil, obtained under our experimental conditions, is similar to or higher than broccoli. Moreover, bróquil contributes to daily mineral intake because the results show high quantities of potassium, calcium, phosphorus, sulfur, and iron. The variability found also makes these landraces an important resource for genetic improvement, especially regarding the HB5 accession, which could be considered a promising candidate for the bróquil-headed type due to the high values for most of the parameters assessed. In conclusion, our results revealed interesting antioxidant properties and mineral composition for this traditional crop, which reinforce the nutritional value of bróquil, enriched in bioactive compounds that promote healthy nutrition.

## Data availability statement

The raw data supporting the conclusions of this article will be made available by the authors, without undue reservation.

## Author contributions

CMo: investigation, data curation, formal analysis, funding acquisition, writing—original draft, supervision, methodology, and writing—review and editing. RZ: investigation, data curation, formal analysis, writing original draft, supervision, methodology, and writing—review and editing. SL: investigation, data curation, formal analysis, and writing the original draft. CMa: investigation, data curation, writing original draft, supervision, and writing—review and editing. All authors have read and agreed to the published version of the manuscript.
